# Use of spectacles for distance vision: coverage, unmet needs and barriers in a rural area of North India

**DOI:** 10.1186/s12886-019-1262-3

**Published:** 2019-12-12

**Authors:** Sumit Malhotra, Mani Kalaivani, Ramashankar Rath, Manya Prasad, Praveen Vashist, Noopur Gupta, Suraj Singh Senjam, Sanjeev Kumar Gupta

**Affiliations:** 1 Centre for Community Medicine, All India Institute of Medical Sciences, New Delhi, India; 2 Department of Biostatistics, All India Institute of Medical Sciences, New Delhi, India; 3 Community Ophthalmology, Dr. Rajendra Prasad Centre for Ophthalmic Sciences, All India Institute of Medical Sciences, New Delhi, India; 4 Dr. Rajendra Prasad Centre for Ophthalmic Sciences, All India Institute of Medical Sciences, New Delhi, 110029 India

**Keywords:** Spectacle coverage, Unmet need, Distance vision, Jhajjar, Rural

## Abstract

**Background:**

Uncorrected refractive errors contribute enormously to the burden of avoidable visual impairment worldwide. There is a huge disparity in different parts of the globe in context to spectacle coverage for distance vision. This study was undertaken with objectives of determining prevalence of spectacle coverage, unmet needs and associated factors among adults in a rural community of north India.

**Methods:**

A community-based cross-sectional study was carried out within selected clusters of Jhajjar district of Haryana. All participants aged > 15 years underwent visual acuity assessment by LogMAR “E” screening chart. Participants with presenting visual acuity < 6/12 in any eye and all current spectacle users underwent detailed ophthalmic examination and refraction. Additional details about spectacles, barriers for their use and willingness to pay for them were collected. Participants with met and unmet need for spectacle use at visual acuity > 6/12 was computed. These are reported as proportions with 95% confidence intervals. Associated factors with unmet need were determined using bivariable and multivariable logistic regression analysis.

**Results:**

A total of 6910 participants were examined. The current spectacle use was 7.5% (95% Confidence Interval CI: 6.5, 8.7). The spectacle coverage was found in 33.3% (95% CI: 30.0, 36.7) participants among those in need. The unmet need was found in 10.8% of participants (95% CI: 10.1, 11.6). On multivariable analysis, odds of unmet need was associated with age, gender, level of education and marriage status. The most common barrier for refractive correction was lack of perceived need for refraction and its correction.

**Conclusion:**

There is substantial unmet need for distance vision spectacles in this population. It is imperative that multi-component intervention be implemented to improve spectacle coverage in this rural north Indian setting.

## Background

Uncorrected refractive errors are recognized globally as the main cause of avoidable visual impairment [1]. It has been estimated worldwide that among the people with moderate or severe visual impairment in year 2015, 116.3 million people are with uncorrected refractive errors, with burden reaching 127.7 million by 2020. Additionally, among people with blindness in year 2015, there are 7.4 million people with uncorrected refractive errors with anticipated increase to 8 million people in 2020 [2]. Majority of these people live in low and middle income countries with south Asian countries having maximum burden in terms of absolute numbers.^2^ Within South Asia that includes India; uncorrected refractive errors contribute 68% of moderate to severe visual impairment and 37% of blindness [2]. Uncorrected refractive errors affect educational prospects, productivity and quality of life [3]. As per an economic analysis, uncorrected refractive errors resulted in 269 International Dollars total global productivity loss [4]. Considering this enormous public health impact, the condition has been included as a priority eye health problem in Vision 2020 initiative [5].

Global efforts are underway to universalize eye care as per World Health Organization agenda plan 2014–2019 [6]. Measurement in terms of utilization of services will be imperative for tracking progress. Spectacle coverage is one such indicator for uncorrected refractive errors highlighting the reach of simple low cost eye care services. As per a global report collating information from 27 countries, the spectacle coverage for distance vision varied from 2 to 93% [7]. There is paucity of available information on this indicator from south Asian region, despite having highest burden. Limited numbers of surveys have been done in India on this issue, largely from southern parts of the country [8–11]. Against this background, we report here findings of an assessment conducted in a rural setting of north India with objectives of determining spectacle coverage, unmet needs for spectacle use and its barriers. Additionally, we also examined factors associated with them.

## Methods

### Study setting and design

This was a community based cross-sectional study conducted in Jhajjar district of Haryana. The population of this district was around 9,00,000 [12]. This study was done in two sub districts (Bahadurgarh and Jhajjar). A list of villages in these sub districts was prepared and arranged in the increasing population size. A sample size of 6913 adults was calculated assuming spectacle coverage of 29% [10] with relative precision of 5%, design effect 1.5, and a non-response rate of 15%.

Selection of villages was done according to Probability Proportionate to Size. A total of thirty-four villages were selected in this study. Villages were considered clusters for this study. Each village was broken down to segments of 400–600 population. One compact segment was selected randomly using the sealed envelopes. All adults aged more than 14 years were enumerated in this segment. The study was conducted from January to May 2014.

### Data collection

The data collection was done by two teams with each team comprising of one optometrist, social worker, and health assistant. All team members were experienced and were running primary care vision clinics for more than 2 years. The teams were sensitized and trained in all procedures related to data collection and examination. Firstly, demographic details were collected by a social worker and a health assistant during their house to house visit. History about distance vision spectacle use was also collected. All participants underwent visual acuity testing using logMAR “E” screening chart corresponding to five 6/12 optotypes. The visual measurement was done at 4 m’ distance, outdoors and in shaded on bright and sunny days. Presenting visual acuity was considered as vision with spectacles if using spectacles for distance vision. Identification of four letters out of five was considered as pass criteria.

All participants with visual acuity less than 6/12 in either eye, using spectacles and those with previous cataract surgery were referred to temporary make shift clinic where optometrists performed detailed eye assessment using logMAR “E” chart and refraction. All those participants where unmet need for spectacles was found by the optometrist, barriers for the need were ascertained. Such participants were also enquired about willingness to pay for spectacles.

Pilot testing was done before commencement of the actual study in an area other than the study villages. Continuous scrutiny of all the data collection procedures by investigating team comprising of epidemiologist and ophthalmologist was performed.

### Operational definitions

These had been used as per studies conducted earlier [13]. The definitions of met and unmet need for uncorrected refractive errors have been kept in mind, the definition of visual impairment (presenting visual acuity in better eye < 6/12) and it is contributed by this cause, confirmed upon subjective acceptance and improvement of visual acuity > 6/12 by the optometrist.

Current spectacle use: Adults who reported using distance vision spectacles at the time of examination.

Met need was considered as those who wore distance vision spectacles and had visual acuity less than 6/12 in the better eye without correction, but who achieved 6/12 or more in the better eye with their present distance vision spectacles.

Unmet need included those persons who were not using distance vision spectacles and the presenting vision of better eye was less than 6/12 and on subjective acceptance the vision of better eye increased to ≥6/12. Unmet need was also considered as those who were using distance vision spectacles and the presenting visual acuity of better eye was less than 6/12 but on subjective acceptance the vision of better eye increased to equal to or better than 6/12.

Spectacle coverage was defined as met need/(met need + unmet need) X 100%

Below Poverty Line (BPL): was considered when monthly income of the family was less than US$4.6 [Indian National Rupees INR 300] and was confirmed by BPL ration card by the family [14].

### Statistical analysis

Data were entered and managed in MS Access 2007 and statistical analysis was carried out using Stata 12.0 (StataCorp LP, 4905 Lakeway Drive, College Station, Texas, USA). The spectacle coverages (along with 95% Confidence Intervals CI) are calculated and reported. Bivariable and multivariable analysis was carried out using logistic regression for complex survey data for determining associated factors with unmet need for spectacles. Both crude Odds Ratio OR and Adjusted Odds Ratio AOR (along with 95% CI) were computed. The *p*-value less than 0.05 was considered statistically significant.

## Results

A total of 7495 adults > 15 years were enumerated and 6910 participants (92%) were examined by the study teams. Their characteristics are shown in Table 1. Majority (41%) of the examined participants belonged to the age category 15 to 29 years and 35% were in age group 30–49 years and rest > 50 years comprised 24% of study participants. Around half of the participants (49%) were men. Of all the participants, 40% were educated up to secondary class level whereas around one third of the participants were educated more than secondary level. Around 47% of participants were involved in housework where as one fifth (21%) of participants were unemployed/students.

**Table 1:** Socio-demographic characteristics of enumerated and examined population

Variable	Enumerated population (*n* = 7495)	Examined Population (*n* = 6910)
Age category (Years)		
15–29	2880 (38.4)	2826 (40.9)
30–39	1493 (19.9)	1422 (20.6)
40–49	1097 (14.6)	972 (14.1)
50–59	771 (10.3)	623 (9.0)
60–69	745 (9.9)	629 (9.1)
70 and above	509 (6.8)	438 (6.3)
Gender		
Men	3845 (51.3)	3407 (49.3)
Women	3650 (48.7)	3503 (50.7)
Education		
Illiterate	1463 (19.5)	1310 (19.0)
Primary (Upto 5th Class)	730 (9.7)	659 (9.5)
Secondary (6th to 10th Class)	3026 (40.4)	2781 (40.3)
Senior Secondary and above	2276 (30.4)	2160 (31.3)
Occupation		
Housework	3411 (45.5)	3225 (46.7)
Labour- Agricultural/ Non-Agricultural	1436 (19.2)	1249 (18.1)
Office/ Skilled work	1112 (14.8)	970 (14.0)
Unemployed/ Students	1536 (20.5)	1466 (21.2)
Poverty line		
Above Poverty Line (APL)	5991 (79.9)	5494 (79.5)
Below Poverty Line (BPL)	1504 (20.1)	1416 (20.5)

### Study flow and current spectacle use for distance vision

**Fig. 1:**
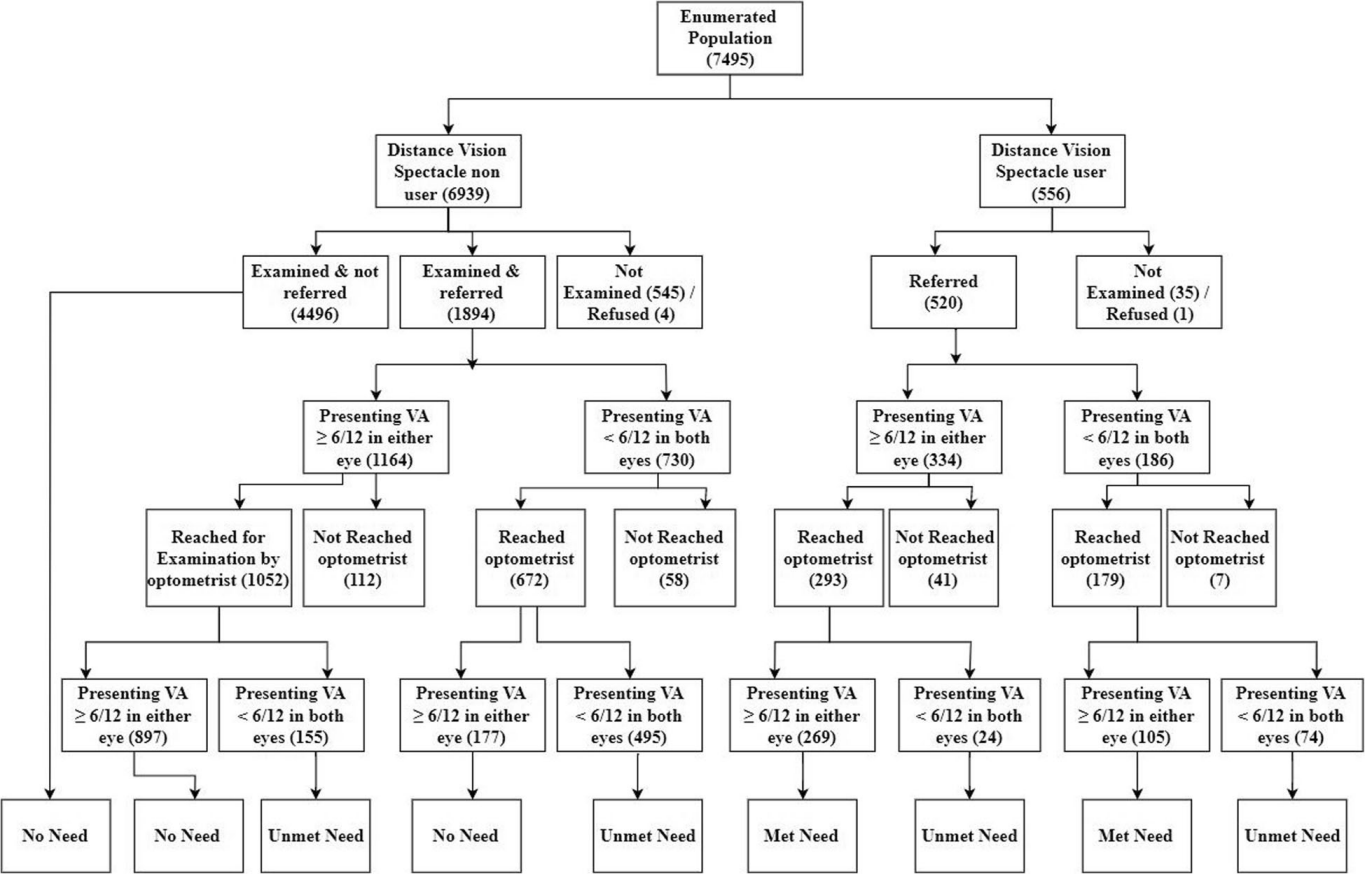
Study flow of participants. Among participants examined, that were spectacle users and non-users, met and unmet need is depicted

The study flow of participants is depicted in Fig. 1. Among all the participants, with no history of current use of distance vision spectacles (6939), a total of 6390 (92%) were examined during house to house survey. Referral was done to optometrist for 1894 participants, out of which, 1724 participants, were assessed by the optometrist again. Overall, in this group of participants combined, no need for spectacles (visual acuity > 6/12 in better eye) was found in 5570 adults.

There were total of 556 adults enumerated with history of current use of distance vision spectacles, out of which 520 participants were examined with a prevalence of current spectacle use as 7.5% (95% CI:6.5,8.7). We could gather additional details about their spectacles in 480 participants and their characteristics are shown in Table 2. Median duration of spectacle use was 58 months with Inter Quartile Range (IQR) of 24 to 96 months. Visual acuity was tested in the private hospital for around 62% of the participants and at government hospitals for 28.7% of them. Spectacles were obtained from a private hospital by 48% and from privately owned optical shop by 30% participants. These were obtained free by 7.3% of users. The median amount paid by participants for a pair of spectacles was INR 300 US$5 (IQR: INR 200–450). Around 86% of the users were satisfied with their current spectacles. From those who were not satisfied (59, 14%), the predominant reason was no improvement of vision followed by headache. Spectacle wearers were referred to the optometrist, and 472 participants’ (91%) reached the clinic for repeat eye assessment.

**Table 2:** Socio-demographic characteristics of spectacle users

Variable	Spectacle Users (*n* = 520)
Age category (Years)	
15–29	111 (21.3)
30–39	37 (7.1)
40–49	37(7.1)
50–59	83 (16.0)
60–69	119 (22.9)
70 and above	133 (25.6)
Gender	
Men	205 (39.4)
Women	315 (60.6)
Education	
Illiterate	173 (19.5)
Primary (Upto 5th Class)	61 (9.7)
Secondary (6th to 10th Class)	166 (40.4)
Senior Secondary and above	120 (30.4)
Occupation	
Housework	316 (60.7)
Labour- Agricultural/ Non-Agricultural	40 (7.7)
Office/ Skilled work	42 (8.0)
Unemployed/ Students	122 (23.5)
Poverty line	
Above Poverty Line (APL)	434 (83.5)
Below Poverty Line (BPL)	86 (16.5)

### Spectacle coverage

The total need for spectacles was found in 1122 participants’ out of all examined adults (16.7%) based on initial visual acuity assessment and later on measurement of subjective acceptance wherever needed. The met need was observed in 374 participants (that is, the refractive errors were corrected by using spectacles by adults on their own). The spectacle coverage of overall study population was found to be 33.3% (95% CI: 30.0, 36.7) among those in need. When segregated, highest proportion of spectacle coverage (75.4%) was found in the age group of 15–29 years (75%); males (35%); married adults (36%); working in office/skilled workers (58%); educated above senior secondary levels (71%) and those belonging to above poverty lines (35%). (Table 3).

**Table 3:** Spectacle coverage and associated factors of unmet need for spectacles by logistic regression (*n* = 1122)

Variable	Met Need (374)	Unmet Need (748)	Spectacle coverage Prevalence (95% CI)	Unadjusted OR (95% CI)	*p* value	Adjusted OR (95% CI)	*p* value
Age category							
15–29	89 (75.4)	29 (24.6)	75.4 (16.3, 32.8)	1.0		1.0	
30–39	27 (57.5)	20 (42.5)	57.5 (41.2, 73.6)	2.3 (0.9, 5.4)	0.06	2.2 (0.8, 6.1)	0.13
40–49	33 (39.8)	50 (60.2)	39.8 (28.2, 51.3)	4.6 (2.4, 8.9)	< 0.001	2.5 (1.1, 5.5)	0.02
50–59	66 (36.1)	117 (63.9)	36.1 (28.1, 44.1)	5.4 (3.4, 8.6)	< 0.001	2.9 (1.7, 4.9)	< 0.001
60–69	88 (24.4)	272 (75.6)	24.4 (17.8, 31.1)	9.5 (5.3, 17.1)	< 0.001	4.2 (1.9, 9.2)	0.001
70 and above	71 (21.5)	260 (78.5)	21.5 (16.7, 26.2)	11.2 (6.3, 20.1)	< 0.001	3.9 (1.8, 8.7)	0.001
Gender							
Men	146 (34.7)	275 (65.3)	34.7 (31.1, 38.2)	1.0		1.0	
Women	228 (32.5)	473 (67.5	32.5 (28.2, 36.8)	1.1 (0.9, 1.4)	0.36	0.7 (0.5, 0.9)	0.02
Marriage							
Married	262 (35.6)	474 (64.4)	35.6 (31.7, 39.6)	1.0		1.0	
Single	112 (29.0)	274 (71.0)	29.0 (24.6, 33.8)	1.4 (1.1, 1.7)	0.02	1.7 (1.3, 2.3)	< 0.001
Occupation							
Housework	225 (29.4)	540 (70.6)	29.4 (25.2, 33.6)	1.0		1.0	
Labour	26 (22.8)	88 (77.2)	22.8 (15.1, 32.8)	1.4 (0.8, 2.4)	0.19	2.0 (1.2, 3.5)	0.01
Office/ Skilled work	33 (57.9)	24 (42.1)	57.9 (45.5, 69.4)	0.3 (0.2, 0.5)	< 0.001	0.7 (0.3, 1.3)	0.12
Unemployed/ Students	90 (48.4)	96 (51.6)	48.4 (40.3, 56.6)	0.4 (0.3, 0.7)	< 0.001	0.8 (0.5, 1.2)	0.23
Education							
Illiterate	98 (17.8)	452 (82.2)	17.8 (14.2, 22.0)	1.0		1.0	
Primary	44 (31.9)	94 (68.1)	31.9 (25.3, 39.3)	0.5 (0.3, 0.7)	< 0.001	0.4 (0.3, 0.7)	0.002
Secondary	137 (45.7)	163 (54.3)	45.7 (40.5, 50.9)	0.3 (0.2, 0.4)	< 0.001	0.3 (0.2, 0.5)	< 0.001
Senior Secondary and above	95 (70.9)	39 (29.1)	70.9 (62.3, 78.2)	0.1 (0.1, 0.2)	< 0.001	0.2 (0.1, 0.3)	< 0.001
Poverty line							
Above Poverty Line (APL)	320 (35.3)	587 (64.7)	35.3 (32.2, 38.4)	1.0		1.0	
Below Poverty Line (BPL)	54 (25.1)	161 (74.9)	25.1 (17.8, 34.3)	1.6 (1.1, 2.5)	0.03	1.5 (0.9, 2.3)	0.09

Occupation: Labour- agricultural/non – agricultural; Education: Primary-up to 5th class, secondary-6th to 10th class and senior secondary and above-11th and above; Poverty line: APL-Above Poverty line and BPL-Below Poverty Line; OR-Odds Ratio and CI-Confidence Interval

### Unmet need for spectacles for distance vision and determinants

From the 472 current spectacle users who were examined by the optometrist, 374 (79.2%) had presenting visual acuity more than equal to 6/12 in either eye (Fig. 1). A total of 98 current spectacle users had their presenting visual acuity in the better eye less than 6/12, with an unmet need (that is, their refractive errors were still uncorrected despite use of the spectacles by adults). Additionally, 650 participants were identified with unmet need not using spectacles (that is, their visual acuity was < 6/12 in better eye and improved to > 6/12 on subjective acceptance), thus the total unmet need in our examined population was found to be in 748 participants with prevalence of 10.8% (95% CI:10.1,11.6).

On multivariable analysis, increasing age was found to have significant higher odds of unmet need (Table 3). Compared to adults aged 15–29 yrs., the odds ratio for unmet need was three times higher for 40–49 yrs. [AOR 2.5, 95% CI: 1.1, 5.5]; 50–59 yrs. [AOR 2.9, 95% CI: 1.7, 4.9] and four times higher for 60–69 yrs. [AOR 4.2, 95% CI: 1.9, 9.2] and > 70 yrs. [AOR 3.9, 95% CI:1.8, 8.7] respectively. Unmet need for spectacles was found to be 30% lower in women compared to men [AOR 0.7 (95% CI:0.5, 0.9)]. It was also found two times more in adults living alone compared to married adults living with spouse [AOR 1.7 (95% CI: 1.3, 2.3)]. Increasing education was significantly found to be protective. Compared to illiterate participants, the odds of unmet need were 40% lower amongst adults educated upto primary level [AOR 0.4 95% CI: 0.3, 0.7]; 30% lower for those educated upto secondary level [AOR 0.3, 95% CI: 0.2,0.5] and 20% lower for educated upto and above senior secondary level [AOR 0.2, 95% CI:0.1,0.3].

### Barriers for spectacle use

**Fig. 2:**
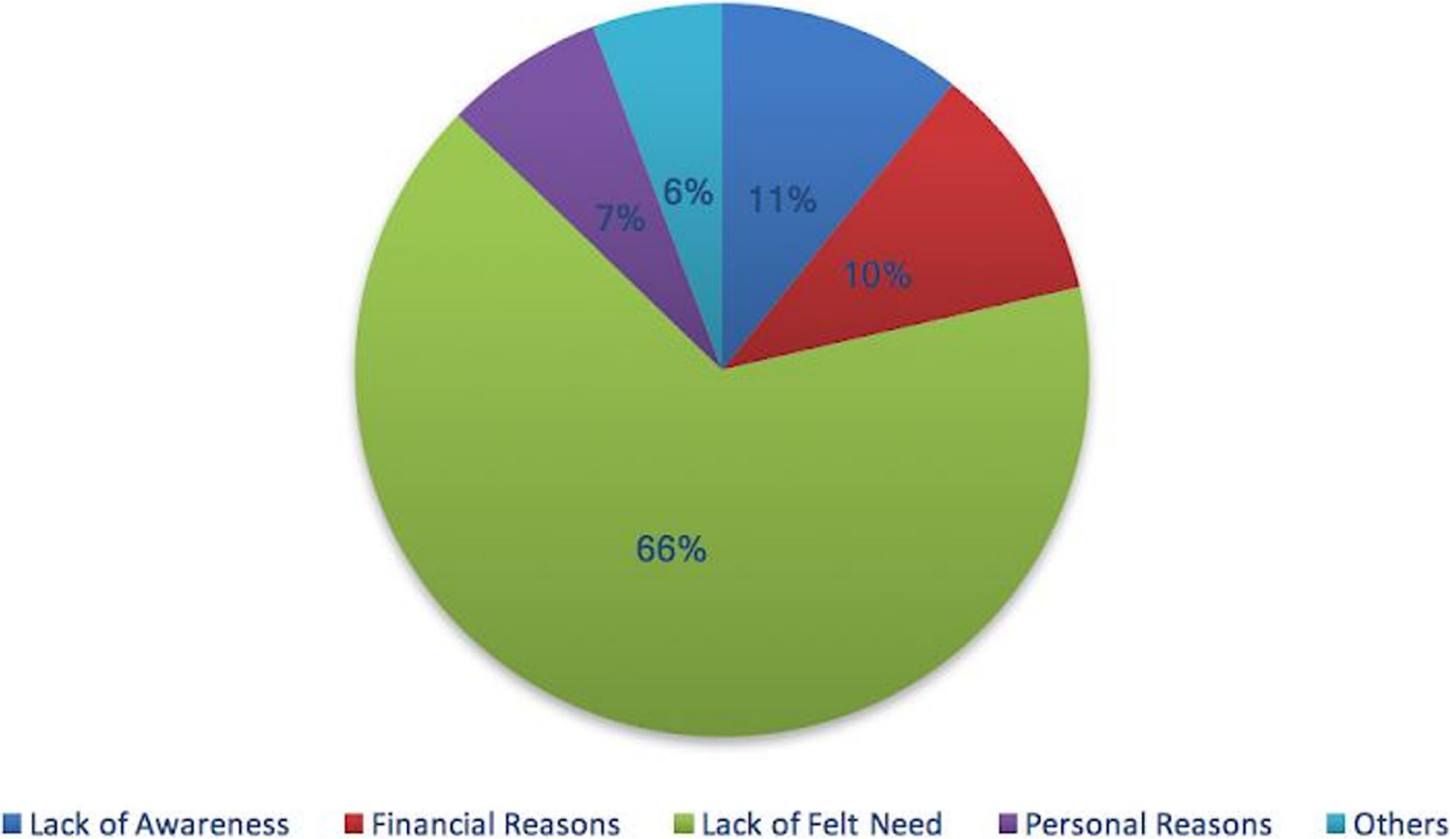
Barriers for spectacle use

Around 64% of the participants with unmet need (480) reported barriers for not getting their refractive correction. 83% of participants (397) did not get their vision examination. Additionally, 5% reported barriers for not buying spectacles after undergoing vision testing. Collectively, lack of felt need (66%), lack of awareness (11%), financial reasons (10%) were most common barriers (Fig. 2). 12% participants reported barriers for not using spectacles after purchase with common reasons as loss, break, difficulty in wearing and no improvement after their use. Participants with unmet need were asked for their willingness to pay for spectacles. 87.5% participants were willing to pay a median cost of US$6 [INR 400 (IQR 200–600)].

## Discussion

The present study was a cross-sectional assessment reporting spectacle use and coverage in a rural north Indian adult population. The prevalence of current spectacle use in our study was 7.5%. This finding was similar to other studies, previously done from India. A study conducted in south India found prevalence of sepctacle use in age group 15–50 years as 7% [95% CI: 6.2, 8%] [9]. In an adult population aged > 30 years in Pakistan, prevalance of spectacle wear was 6.2% [13]. Not many studies have reported the amount spent by people in purchase of spectacles. Our finding is similar to other Indian study that reported more than 80% of participants paid <INR 600 [10US $] for purchase of spectacles [10].

We found the spectacle coverage to be 33.3%. In the age group of 40 years or more it was found to be still less (27%). This was lower than from a previously reported coverage of 54% from southern state of Telangana in India [10] and more than the coverage (20%) reported among adults aged > 40 years an urban area of north India [15] and among adults aged 15–49 years in a south Indian setting where it was reported as 29% [9]. Spectacle coverage reported in specific groups of people within south Indian states was slightly higher than the current study as in marine fishing communities aged > 40 yrs. was 38% [16] and adults aged > 50 yrs. from residential care 35% [17]. These differences could be due to varying study setting, access to health services and differences among the participants studied and varying visual acuity definitions for spectacle coverage. Internationally, also spectacle coverage varies to a greater extent across different settings owing to differences in study methodology, use of definitions, age groups of included participants and health system differences. The spectacle coverage reported in a study from Nigeria was 3% [18], Pakistan 15% [13], Los Angeles 21% [19], Eritrea 22% [20], Bangladesh 25% [21], and China 44% [22].

In this study, the unmet need of refractive error correction was found to be 10.8% of the population covered. This was found to be higher than that reported in adults 15–49 years 4.4% Mahabubnagar district, Andhra Pradesh [9]; slightly lower (11.5%) in adults > 40 yrs. marine fishing communities, Prakasam district, Andhra Pradesh [16] ^16^ and adults > 50 yrs. residential care settings (15%), Andhra Pradesh [17]. These differences might be attributable to differences in study subjects, methodology and setting. The unmet need reported in global studies was Nigeria 9.1% [18], Pakistan 9.4% [13]. Slight differences might be ascribed to varying age groups included in the study, participants and access to health services. We also found amongst participants wearing spectacles, having unmet need of 19%. This was less as reported from a study from Pakistan 26% indicative of certain fractions of spectacle wearers with incorrect prescriptions [13]. It was found that age, gender, marital status and education were associated with unmet need for spectacles. These factors concur with earlier reported studies on unmet need for refractive correction. For each decade, increase in age above 30–39 years, odds of unmet need increased by 1.5 (95% CI: 1.3, 1.7) [13]. It was also reported from Bangladesh, that illiterate adults were significantly more likely to have uncorrected refraction than literate individuals OR 22.5 (95% CI: 14.5, 34.9). The same study also reported that those who had not progressed to secondary school education and above were more likely to have uncorrected refraction than these levels of education [21]. All these factors influence the health-seeking behavior of an individual. Illiteracy might adversely affect the access to health care services and the knowledge regarding how to obtain it. Although some services are provided at no cost, the indirect expenses such as lost wages, travel and other incidental expenses might pose an economic hurdle for uptake of services. Marmamula et al. found age, education and gender to be associated with spectacle coverage among elderly [8]. In another Indian study, spectacle use was significantly higher and positively associated with literacy and employment in the urban population [23].

We observed that most common cause for not doing vision checkup was lack of perceived need. Majority of participants also did not perceive difficulty in working without spectacles and thus did not purchase spectacles. The most common cause for not using spectacles after purchasing was broken glasses in our study. In another setting among elderly, lost spectacles was the most common cause followed by broken glasses [8]. The two most common barriers reported for correction of refractive error are lack of awareness/ perceived need and economic reasons [24, 25]. In a north Indian study from urban areas, lack of awareness emerged to be most important barrier amongst those not availing refractive error services [26]. Another study reported cause of discontinuation of spectacle use as the feeling of discomfort with the prescribed spectacles [27].

Our study has programmatic implications. Extrapolating high unmet need to rural areas of Jhajjar with population size of 0.7 million, there will be 52,088 adults aged 15 years and above with unmet need for spectacles. These adults need to be reached with adequate health system response for detection and correction of refractive errors. The study also identified lack of perceived need as the main barrier in meeting the needs of the target population. This ‘attitude-related’ barrier would pose a challenge to health care providers as it would entail requirement of greater behavioral change efforts. There was a definite gap between the professionally determined need and the perceived need of the participants. This requires intensive behavior change communication efforts to generate sufficient demand among adults, keeping in mind the barriers enlisted through the study participants. Our study highlighted that participants are willing to pay for the spectacles, and thus the health system should address this need appropriately through its services.

Our study has certain strengths. It was one of first assessment undertaken in district Jhajjar within Haryana state, generating necessary evidence for planning and would serve as baseline study for future assessments. The study included a large community based sample and achieved high response rate of 92.2%. The study adopted a rapid assessment methodology as utilized in other settings for examining spectacle coverage and utilizes less resources compared to classic epidemiological studies. Our study has certain limitations. The study was carried out only in rural areas, so our results are not generalizable to urban areas. Also, the definitions used in the study for computing spectacle coverage and unmet need was based on visual acuity (presenting vision < 6/12 in better eye), and subsequently with refractive correction getting > 6/12 in better eye, some participants might be missed who might have lower visual acuity than this but could benefit from spectacle correction. This was based on assumption that adults with vision > 6/12 would face minimal difficulty in their daily activities with limited need and would cover adults with distance visual impairment that could be predominantly due to uncorrected refractive errors. Also, this article doesn’t report presbyopia and related need for near vision spectacles. This is planned for reporting separately. Also, the age group in the current study was broader and not all required presbyopic correction. We have exclusively considered all adults for distance visual impairment due to uncorrected refractive errors in this paper and related unmet need. This rapid assessment approach has been widely utilized in many settings now.

## Conclusions

There is high level of unmet need for spectacles in adults with 33% of spectacle coverage in rural setting of Jhajjar, north India. Lack of perceived need for refractive correction emerged as main barrier impeding uptake of services. Augmentation of eye care services within health systems providing refractive services and spectacles will be imperative in reducing burden of uncorrected refractive errors.

## Data Availability

All data generated or analyzed during this study are included in this published article.

## Abbreviations

AOR: Adjusted Odds Ratio
BPL: Below Poverty Line
CI: Confidence Interval
INR: Indian National Rupees
IQR: Inter Quartile Range
